# Myeloid-specific fatty acid transport protein 4 deficiency induces a sex-dimorphic susceptibility for nonalcoholic steatohepatitis in mice fed a high-fat, high-cholesterol diet

**DOI:** 10.1152/ajpgi.00181.2022

**Published:** 2023-03-07

**Authors:** Deniz Göcebe, Chutima Jansakun, Yuling Zhang, Simone Staffer, Sabine Tuma-Kellner, Sandro Altamura, Martina U. Muckenthaler, Uta Merle, Thomas Herrmann, Walee Chamulitrat

**Affiliations:** ^1^Department of Internal Medicine IV, University Hospital Heidelberg, Heidelberg, Germany; ^2^School of Allied Health Sciences, Walailak University, Nakhonsrithammarat, Thailand; ^3^Department of Pediatric Oncology, Hematology and Immunology, University Hospital Heidelberg, Heidelberg, Germany; ^4^Translational Lung Research Center Heidelberg, German Center for Lung Research (DZL), German Centre for Cardiovascular Research, Partner Site, University of Heidelberg, Heidelberg, Germany; ^5^Westkuesten Hospital, Heide, Germany

**Keywords:** fatty acid transport proteins, high-fat, high-cholesterol diets, macrophage polarization, monocyte chemoattractant protein-1, nonalcoholic steatohepatitis

## Abstract

Newborns with FATP4 mutations exhibit ichthyosis prematurity syndrome (IPS), and adult patients show skin hyperkeratosis, allergies, and eosinophilia. We have previously shown that the polarization of macrophages is altered by FATP4 deficiency; however, the role of myeloid FATP4 in the pathogenesis of nonalcoholic steatohepatitis (NASH) is not known. We herein phenotyped myeloid-specific Fatp4-deficient (Fatp4^M−/−^) mice under chow and high-fat, high-cholesterol (HFHC) diet. Bone-marrow-derived macrophages (BMDMs) from Fatp4^M−/−^ mice showed significant reduction in cellular sphingolipids in males and females, and additionally phospholipids in females. BMDMs and Kupffer cells from Fatp4^M−/−^ mice exhibited increased LPS-dependent activation of proinflammatory cytokines and transcription factors PPARγ, CEBPα, and p-FoxO1. Correspondingly, these mutants under chow diet displayed thrombocytopenia, splenomegaly, and elevated liver enzymes. After HFHC feeding, Fatp4^M−/−^ mice showed increased MCP-1 expression in livers and subcutaneous fat. Plasma MCP-1, IL4, and IL13 levels were elevated in male and female mutants, and female mutants additionally showed elevation of IL5 and IL6. After HFHC feeding, male mutants showed an increase in hepatic steatosis and inflammation, whereas female mutants showed a greater severity in hepatic fibrosis associated with immune cell infiltration. Thus, myeloid-FATP4 deficiency led to steatotic and inflammatory NASH in males and females, respectively. Our work offers some implications for patients with FATP4 mutations and also highlights considerations in the design of sex-targeted therapies for NASH treatment.

**NEW & NOTEWORTHY** FATP4 deficiency in BMDMs and Kupffer cells led to increased proinflammatory response. Fatp4^M−/−^ mice displayed thrombocytopenia, splenomegaly, and elevated liver enzymes. In response to HFHC feeding, male mutants were prone to hepatic steatosis, whereas female mutants showed exaggerated fibrosis. Our study provides insights into a sex-dimorphic susceptibility to NASH by myeloid-FATP4 deficiency.

## Graphical abstract

**Figure:**
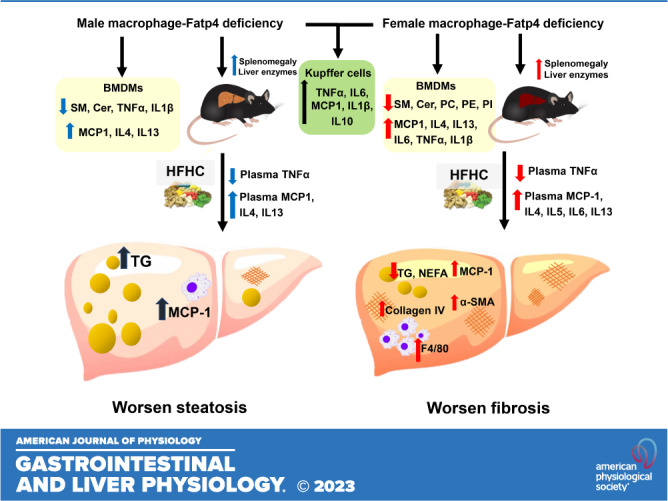

## INTRODUCTION

Fatty acid transport protein 4 (FATP4, SLC27A4) belonging to solute-carrier 27 A families is an acyl-coenzyme A synthetase that activates long-chain and very long-chain fatty acids (VLFAs) by thioesterification (1). The product acyl-CoenzymeAs (acyl-CoAs) is utilized or directed for specific metabolic pathways, such as the syntheses of triglycerides (TGs) and structural lipids including phospholipids (PLs) and sphingolipids (SPLs), as well as elongation, desaturation, and degradation of fatty acids (FA) (1, 2). Acyl-CoAs also participate in protein acylation and mediate signaling and regulation of intracellular functions (2). Inactivation of FATP enzymes in invertebrates leads to development of cardiomyopathy, retinal degeneration, fat storage disease, and dermopathies (3). FATP4 inactivation in vertebrates has led to abnormalities in skin with features of restrictive dermopathies as reported in global (4–6) and keratinocyte-specific (7) Fatp4-deficient mice. Skin abnormalities are also seen in patients with FATP4 mutations including newborns (8) with ichthyosis prematurity syndrome (IPS) and adults (9, 10) with a rare autosomal recessive disorder. The loss of skin permeability barrier in Fatp4-null mice is due to a decrease in keratinocyte ceramides (Cer) containing VLFAs (4) and ultra-VLFAs including barrier lipids, omega-*O*-acylceramides and omega-hydroxyceramides (11, 12). This indicates the specificity of FATP4 on Cer synthesis and metabolism in the skin. A metabolic shift toward neutral lipids, such as TGs, has also been reported in skin fibroblasts (13) and skin (4, 12) of Fatp4-null mice. This metabolic shift toward TGs leads to exacerbated adiposity and hepatic steatosis in adipose-specific Fatp4-deficient mice fed with high-fat diet (14). The elevation of blood lipids was also observed in liver-specific, Fatp4-deficient mice fed with high-sugar and high-fat diets (15). These results are consistent with an association of FATP4 with adiposity, insulin resistance, and blood lipids in general populations (16–18). However, the role of FATP4 on the development of nonalcoholic steatohepatitis (NASH) by modulating inflammation is largely unknown.

As adult patients with FATP4 mutations manifest allergies and eosinophilia (9), we therefore have been investigating the role of FATP4 in myeloid cells including macrophages (MΦ) since FATP4 is expressed in human monocytes (19), human monocytic leukemia THP-1 cells (20), and mouse bone-marrow-derived macrophages (BMDMs) (21). We have shown that BMDMs from male myeloid-specific Fatp4-deficient (Fatp4^M−/−^) mice showed a decrease in Cer containing VLFAs associated with an attenuation of proinflammatory TNFα at basal conditions and during tunicamycin-induced stress (22). We hypothesized that such alteration in MΦ polarization may render an alteration in metabolic response during NASH, we therefore aimed to investigate whether these Fatp4^M−/−^ mice could show phenotypic alterations in cultured MΦ and during NASH in vivo. Herein, NASH was induced by feeding mice with atherogenic Paigen high-fat, high-cholesterol (HFHC) diet because cholesterol accumulation in MΦ leads to formation of foam cells (23) which contributes to NASH (24). We here demonstrated that myeloid-FATP4 deficiency led to alterations in inflammatory cytokines and transcription factors in BMDMs and Kupffer cells in vitro. Fatp4^M−/−^ mice fed with HFHC showed exaggeration of hepatic steatosis in males and hepatic inflammatory fibrosis to a greater extent in females. Thus, our study highlighted a metabolic control on immune response by myeloid-FATP4 deletion, which plays a pivotal role in the development of NASH in a sex-dimorphic manner.

## EXPERIMENTAL PROCEDURES

### Generation of Fatp4^M−/−^ Mice

Myeloid-specific Fatp4-deficient (Fatp4^M−/−^) mice were generated by interbreeding floxed Fatp4-allele (Fatp*^flox/wt^*) mice (7, 14) with *LysM-Cre* transgenic mice to generate Fatp*^flox/wt^ LysM-Cre^Tg/0^* double-mutant mice carrying a floxed Fatp4 allele and the *LysM-Cre* transgene. Fatp4*^flox^LysM-Cre^Tg^* mice were mutants with myeloid-specific deletion of exon 3 in the *Fatp4* gene. Mice were backcrossed at least 20 generations on the C57BL/6 background. Control (con) were Fatp*^flox/wt^* (Flox) and WT C57BL/6 mice for in vitro and in vivo experiments, respectively. All mice were bred and maintained at the animal facility of the University of Heidelberg, Im Neuenheimer Feld 347.

For genotyping, genomic DNA was isolated from tail biopsies and Fatp4 genotyping was performed by PCR using the following primer pairs. For Fatp4*^wt^* (350 bp amplicon) and Fatp4*^flox^* (430 bp amplicon), primers were 5′-GAGCTTCTATGGCAGTGAGG-3′ and 5′-GAAGCTATCAGTGCTAAGCC-3′. PCR conditions for Fatp4 were 1 × 94°C, 5 min; 35 × 94°C 30 s, 55°C 30 s, 72°C 30 s, and 1 × 72°C 5 min. For lysM-Cre*^0^* for wild-type (WT; 350 bp amplicon), primers were 5′-CTTGGGCTGCCAGAATTTCTC-3′ and 5′-TTACAGTCGGCCAGGCTGAC-3′. For lysM-Cre*^Tg^* (700 bp amplicon), primers were 5′-CTTGGGCTGCCAGAATTTCTC-3′ and 5′-CCCAGAAATGCCAGATTACG-3′. PCR conditions for lysM-Cre were: 1 × 94°C 1.5 min; 10 × 95°C 30 s, 65°C 30 s, 72°C 30 s, −1.5°C each cycle; 26 × 94°C 30 s, 60°C 30 s, 72°C 30 s; and 1 × 72°C 5 min.

### Animal Treatment

For feeding experiments, male and female control and Fatp4^M−/−^ mice (8–10 mice per group) at 12 mo of age were fed with Paigen HFHC diet (containing 15% fat, 1.25% cholesterol, and 0.5% sodium cholate, Cat. No. E15104-3474 from ssniff Spezialdiäten, Soest, Germany) for 16 wk. Chow (containing 5.3% fat, LASQC diet Rod 18-A from LASvendi, Soest, Germany) was used as a control diet. The feeding of mice took place at the animal facility of the University of Heidelberg. For HFHC feeding cohort, after 4 h starvation mice were euthanized by carbon dioxide, and liver, spleen, and blood were harvested. Liver samples were fixed in 10% neutral buffered formalin or stored in RNAlater (Sigma-Aldrich, Taufkirchen, Germany). Liver and subcutaneous fat (inguinal) tissues were snap-frozen and kept at -80°C. In another cohort to investigate LPS response, control and Fatp4^M−/−^ mice were intraperitoneally injected with saline or *Escherichia coli* lipopolysaccharide (LPS, O111:B4, Sigma-Aldrich) at 1 mg/kg for males and 0.5 mg/kg for females. After overnight food deprivation, they were euthanized for collection of blood and measurement of complete blood counts. The procedures of mouse feeding and treatment were approved by University of Heidelberg Institutional Animal Care and Use Committee and the German Authority (Baden-Württemberg Regierungspräsidium Karlsruhe) with license number 35–9185.81/G248/11, according to Animal Welfare Laboratory Animal Ordinance from the German Animal Welfare Act.

### Preparation and Treatment of BMDMs

Control and Fatp4^M−/−^ mice at ∼7–10 mo old were used for BMDM preparations. After euthanasia, bone marrow cells were harvested by centrifugation of bone marrow cells from femurs and tibias according to a published protocol (25). Bone marrow cells were plated in a 6-well plate at 1.5 × 10^6^ cells/well and cultured in RPMI 1640 containing 20 ng/mL recombinant murine granulocyte-macrophage colony-stimulating factor (PeproTech, Hamburg, Germany), 10% FBS, penicillin, and streptomycin. Fresh complete medium was replaced every 3 days for 10 days. The obtained BMDMs were subsequently treated with PBS or 100 ng/mL *E. coli* (O111:B4) LPS (Sigma-Aldrich) for 24 h. BMDM supernatants and cell lysates were collected for analyses.

### Preparation and Treatment of Kupffer Cells

Control and Fatp4^M−/−^ mice at ∼13 mo old were used for preparations of Kupffer cells. Kupffer cells were prepared by gradient separations using Optiprep (60% iodixanol, Progen, Heidelberg, Germany) according to a published procedure (26, 27). Briefly, after anesthesia mouse liver was perfused to remove blood followed by perfusion with buffer containing collagenase type 2 (CLS-2, Worthington, Biochem Corp., NJ). After centrifugation at 20 *g* for 2 min, the collected supernatants were centrifuged at 800 *g*, and the pellets were resuspended in RMPI 1640 and aliquoted in 12-mL tubes. After 800 *g* centrifugation, the pellets were resuspended in 2-mL 24% iodixanol and overlaid with 2 mL of 17%, 11.5%, 8.4%, and 0% iodixanol in RPMI1640. After centrifugation at 1,400 *g* for 20 min, Kupffer cells were collected from the 11.5/8.4% interphase and resuspended in RPMI 1640. Cells were plated in a 96-well plate at 2 × 10^5^ cells per well and cultured in a 5% CO_2_ incubator at 37°C. On the next day, Kupffer cells were treated with PBS or *E. coli* LPS (Sigma) at 100 ng/mL for 24 h. The medium and cells were harvested and stored at −80°C. Kupffer cells from a control and a Fatp4^M−/−^ mouse were prepared on the same day, and the presented results were reproducible from 2–3 preparations. The procedure of liver perfusion in mice was approved by Animal Care and Use Committee of the University of Heidelberg and the German Authority (Baden-Württemberg Regierungspräsidium Karlsruhe) with a license number 35–9185.81/A13/18.

### Profiling of PLs and SPLs by LC/MS-MS

Lipids from BMDMs were extracted by using hexane:isopropanol (3:2 vol/vol) containing internal standards according to our published procedure (22). The profile of 164 phospholipids (PLs) and sphingolipids (SPLs) was obtained by liquid-chromatography mass spectrometry (LC-MS/MS) with running conditions described previously (22). Internal standards were phosphatidylcholine (PC)-17:0/17:0, phosphatidylethanolamine (PE)-12:0/12:0, phosphatidylserine (PS)-17:0/17:0, phosphatidylinositol (PI)-17:0/17:0, and Cer-17:0 (Avanti Polar Lipids, Alabaster, AL). Internal standard peak areas were monitored for quality control and used for quantification of analytes of samples and standards. Data acquisition and processing were performed with Masslynx version 4.1 software. The data were exported to Excel sheets and analyte/internal standard ratios were used to determine the response in arbitrary units, which were normalized to cellular milligrams protein.

### Clinical Chemistry

After euthanasia of mice, EDTA-treated blood was subjected to determination of complete blood counts using a scil Vet abc Plus+ hematology analyzer (Scil animal care company GmbH, Viernheim, Germany). Plasma alanine transaminase (ALT) and lactate dehydrogenase (LDH) activities were determined with Randox kits (Krefeld, Germany). Liver homogenates were subjected to lipid extraction according to a published method (28). TGs and nonesterified fatty acids (NEFA) in serum and liver lipid extracts were determined by using LabAssay TAG and NEFA-HR kits (Wako Chemicals, Neuss, Germany), respectively. Cholesterol (CHOL) and PLs were determined by cholesterol assay kits (Randox) and phospholipid assay kits (Mti-diagnostics, Idstein, Germany), respectively.

### ELISA

The levels of mouse cytokines IL1β, IL4, IL5, IL6, IL13, IL10, TNFα, and MCP-1 were determined in supernatants of cultured MΦ and mouse plasma samples using murine standard ABTS ELISA development kits from PeproTech.

### Histology and Immunohistochemistry

The preparation of paraffin blocks of livers, sectioning to 3-µm liver slides, and the staining with hematoxylin and eosin (H&E) were carried out at the Center for Model System and Comparative Pathology, Institute of Pathology, University Hospital Heidelberg, Im Neuenheimer Feld 247. For Sirius-red staining of collagen, paraffin liver sections were stained with 0.1% Sirius-red (Waldeck, Münster, Germany) according to standard protocols. For immunohistochemistry (IHC) staining, liver slides were deparaffinized and subjected to heat-mediated antigen retrieval in citrate buffer pH 6. After H_2_O_2_ treatment and blocking with goat serum, liver slides were incubated with a primary rabbit antibody against F4/80 (SP115, ab111101, Abcam, 1:100 dilution), αSMA (E184, ab32575, Abcam, 1:250 dilution) or collagen IV (ab6586, Abcam, 1:250 dilution) at 4°C overnight. For collagen IV IHC, liver slides were permeabilized with 0.1% Triton X-100 in PBS for 1 h before serum blocking. Rabbit-specific HRP/DAB (ABC) IHC kit (ab64261, Abcam) was used for detection. Pictures were taken by an Olympus IX50 microscope. Histological hepatic steatosis, Sirius-red (+), F4/80 (+), αSMA (+), and collagen IV (+) areas were analyzed from 10–25 pictures per slide using Adobe Photoshop CS2 v. 9.0.

### Gene Expression

RNA was isolated using an RNeasy Mini Kit and RNase-Free DNase Set (Qiagen, Hilden, Germany) according to instructions. RNA concentrations were determined with a NanoDrop 2000 spectrophotometer (Thermo Fisher Scientific). RNA was reversed transcribed using a FastGene Scriptase Basic kit (Nippon Genetics Europe, Düren, Germany). Gene expression was determined by quantitative real-time polymerase-chain-reaction performed on an Applied Biosystems 7500 System (Thermo Fisher Scientific) using TaqMan Gene expression assays. The target gene expression was calculated using comparative Ct (ΔΔCt) method and normalized to the housekeeping gene GAPDH.

### Western Blotting

Liver (40 mg) or subcutaneous fat (100 mg) was homogenized using a Bullet Blender. Lysates from cultured MΦ (30 µg), liver homogenates (30 µg), or visceral fat homogenates (80 µg) were separated by gel electrophoresis and transferred onto nitrocellulose or PVDF membranes. Membranes were incubated overnight with an antibody against SLC27A4/FATP4 (ab200353, Abcam) or CEBPα (clone EP709Y, Cat. No. 1704-1, Epitomics). Primary antibodies purchased from Cell Signaling (Frankfurt, Germany) were antibodies against MCP-1 (Cat. No. 2029), PPARγ (Cat. No. 2435), p-FoxO1 (Ser256; Cat. No. 9461), ACC (Cat. No. 3662), FASN (C20G5, Cat. No. 3180), and GAPDH (14C10, Cat. No. 2118). After incubation with a secondary HRP-linked antibody, proteins were visualized by using Luminata Forte Western HRP Substrate (Millipore, Darmstadt, Germany). Quantification of proteins was carried out using ImageJ after normalization to GAPDH.

### Statistical Analyses

Results were presented as means ± SE. Statistical significance analyses were analyzed by Mann-Whitney *U* tests for paired comparisons and Kruskal-Wallis tests with Dunn’s selected pair posttests for multiple comparisons. *P* < 0.05 was considered significant.

## RESULTS

### Verification and Initial Characterization of White Blood Cells and Lipids in BMDMs of Fatp4^M−/−^ Mice

**Figure 1.**
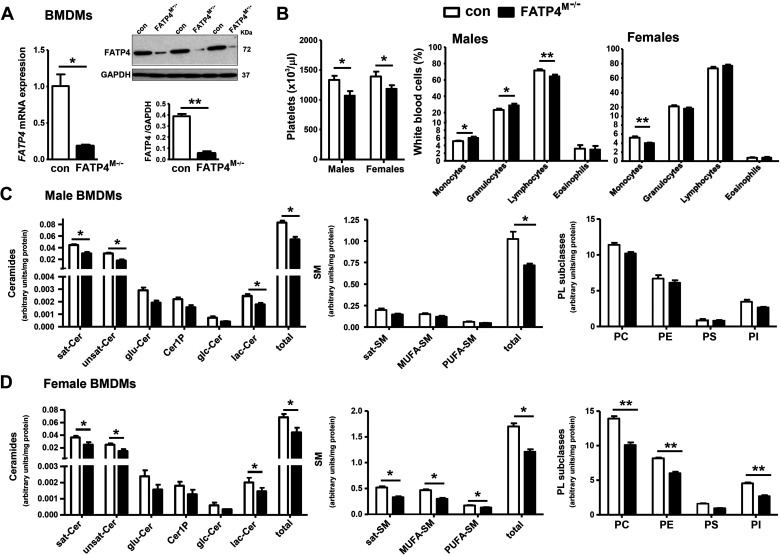
Genotypes and characteristics of white blood cells and BMDMs from control and Fatp4^M−/−^ mice. Control (Con) and Fatp4^M−/−^ mice at 7–10 mo old were used for these analyses. *A*: expression *Fatp4* mRNA and FATP4 protein in BMDMs from male Con and Fatp4^M−/−^ mice. *B*: the absolute counts of blood platelets (×10^3^/µL) and composition (%) of blood monocytes, granulocytes, lymphocytes, and eosinophils from male and female Con and Fatp4^M−/−^ mice. Lipidomics of ceramides (Cer), sphingomyelin (SM), and PL subclasses (PC, PE, PS, and PI) in BMDMs from male (*C*) and female (*D*) Con and Fatp4^M−/−^ mice. Lipidomics data were reported as arbitrary units/mg protein. SM species were reported as saturated-SM, monounsaturated (MUFA-SM), polyunsaturated (PUFA-SM), and total SM. Cer species were reported as saturated-Cer, unsaturated Cer, glucosyl-Cer (glu-Cer), Cer-1-phosphate (Cer1P), galactosyl-Cer (glc-Cer), lactosyl-Cer (lac-Cer), and total Cer. Data are means ± SE, *n* = 4–6 (*A*, *C*, and *D*) and *n* = 5–10 (*B*). *P < 0.05 and **P < 0.01 by Mann-Whitney *U* tests. BMDMs, bone-marrow-derived macrophages; PC, phosphatidylcholine; PE, phosphatidylethanolamine; PI, phosphatidylinositol; PS, phosphatidylserine.

A mouse model of myeloid-specific Fatp4 deficiency was generated and referred to as the Fatp4^M−/−^ mouse. The deletion was verified by a marked reduction of *Fatp4* mRNA and FATP4 protein in BMDMs of Fatp4^M−/−^ mice (Fig. 1*A*). Since blood immune cells arise from hematopoietic progenitor cells in the bone marrows (29), we therefore measured white blood cells from control (Con) and Fatp4^M−/−^ mice. Both male and female mutants showed a decrease in absolute numbers of blood platelets (Fig. 1*B*) indicating induction of thrombocytopenia by the deficiency. For male mutants, the composition of lymphocytes was decreased whereas that of monocytes and granulocytes was increased. On the contrary, female mutants showed a decrease in the composition of monocytes. Thus, myeloid-FATP4 deficiency may have an effect on hematopoietic bone-marrow cells resulting in thrombocytopenia and sex-dependent changes in white blood cell composition.

Our previous lipidomics data revealed a marked reduction in Cer containing VLFAs, such as Cer d18:1/24:1 and Cer d18:1/24:0 in BMDMs from male Fatp4^M−/−^ mice (22). Thus, FATP4 in BMDMs may provide acyl-CoAs for the synthesis VLFA-Cer species, which was also previously observed in keratinocytes (4). Here, LC-MS/MS was utilized to profile subclasses of sphingolipids (SPLs) and phospholipids (PLs) in BMDMs from male (Fig. 1*C*) and female (Fig. 1*D*) Con and Fatp4^M−/−^mice. Among SPLs, both male and female mutants showed a significant decrease in saturated and unsaturated Cer species by ∼28% and total sphingomyelin (SM) by ∼44%. Notably, the decrease in saturated and unsaturated SM was more prominent in female compared with male mutants (Fig. 1*D*). Female mutants similarly showed a significant decrease in phosphatidylcholine (PC), phosphatidylethanolamine (PE), and phosphatidylinositol (PI) by ∼30%. Only a trend decrease in these PLs was observed in male mutants. Thus, myeloid-FATP4 provided acyl-CoAs for the synthesis of Cer species in both males and females, and with preferential synthesis of PLs in females. Taken together, myeloid-FATP4 deficiency led to thrombocytopenia in males and females. The deficiency altered white blood cell composition and the levels of BMDM SPLs and PLs in a sex-dependent manner.

### BMDMs from Fatp4^M−/−^ Mice Show Changes in Inflammatory Cytokines and Transcription Factors

**Figure 2.**
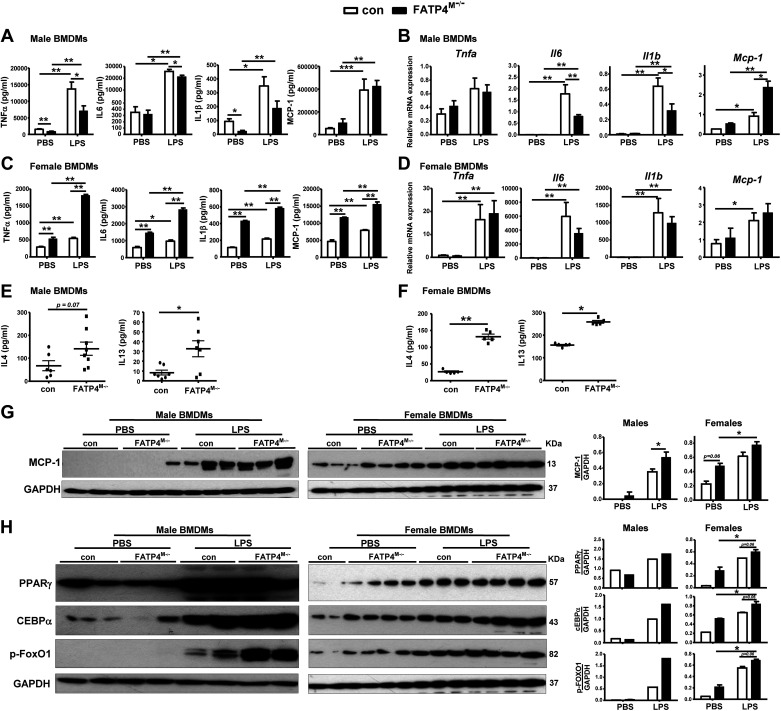
BMDMs from Fatp4^M−/−^ mice show changes in inflammatory cytokines and transcription factors in vitro. BMDMs from male and female control (Con) and Fatp4^M−/−^ mice were treated with PBS or 100 ng/mL LPS for 24 h. The release (pg/mL) of TNFα, IL6, IL1β, and MCP-1 (*A*) and relative mRNA expression of *Tnfa, Il6, Il1b*, and *Mcp-1* (*B*) in PBS- or LPS-treated BMDMs from male mice. The release (pg/mL) of TNFα, IL6, IL1β, and MCP-1 (*C*) and relative mRNA expression of *Tnfa, Il6, Il1b*, and *Mcp-1* (*D*) by PBS- or LPS-treated BMDMs from female mice. The release (pg/mL) of IL4 and IL13 by PBS-treated BMDMs from male (*E*) and female (*F*) mice. Western blot analysis (*left*) and quantification (*right*) of MCP-1 (*G*) and PPARγ, CEBPα, and p-FoxO1 (*H*) in PBS- or LPS-treated BMDMs from male and female mice. Data are means ± SE, *n* = 4–8 (*A***–***D*) and *n* = 2–4 (*G* and *H*). *P < 0.05, **P < 0.01, and ***P < 0.001 by Mann-Whitney or Kruskal-Wallis tests. LPS, lipopolysaccharide. BMDMs, bone-marrow-derived macrophages.

It is shown that Cer (30) and particularly those containing VLFAs (31) are protective by negatively regulating LPS-induced TNFα production. As mutant BMDMs showed a decrease in Cer species (Fig. 1, *C* and *D*), it is anticipated that these cells would show an increase of TNFα. An attenuation of TNFα was previously observed in BMDMs from male mutants (22), we therefore analyzed not only TNFα but also other inflammatory cytokines. Here, BMDMs from male mutants also showed a decrease in TNFα and IL1β release at basal conditions (Fig. 2*A*). On LPS stimulation, these mutant BMDMs showed attenuation of TNFα and IL6 release. Although these cells showed attenuated mRNA expression of *Il6* and *Il1β*, however, they showed further upregulated expression of monocyte chemoattractant protein-1 (*Mcp-1*; Fig. 2*B*). On the contrary, BMDMs from female mutants showed a significant increase in TNFα, IL6, IL1β, and MCP-1 release with or without LPS treatment (Fig. 2*C*). This increase was however not accompanied by mRNA expression of these genes (Fig. 2*D*). Besides proinflammatory cytokines, mutant BMDMs also showed an activation of alternatively activated cytokines IL4 and IL13 in both males (Fig. 2*E*) and females (Fig. 2*F*). Hence, myeloid-FATP4 deficiency in BMDMs stimulated basal IL4 and IL3 in both males and females as well as inflammatory LPS response with a greater extent in females than males.

We further analyzed MCP-1 protein expression in BMDMs to compare inflammatory responses among males and females. At basal conditions, a trend increase in MCP-1 expression was observed in both male and female mutants (Fig. 2*G*). On LPS stimulation, a further increase was also observed with a significant and a trend increase in male and female mutants, respectively. To determine the metabolic control of MΦ cytokine production, we further analyzed the transcription factors peroxisome proliferator-activated receptorγ (PPARγ) and CAAT/enhancer binding protein α (CEBPα) reported to regulate MΦ phenotypes (32) with LPS-dependent (33) and alternative MΦ (34, 35) activation. We also analyzed protein expression of forkhead box protein O1 (FoxO1) reported to regulate Toll-like receptor 4 genes (36) and alternatively activated MΦ (37). At basal conditions, female mutants showed greater activation of PPARγ, CEBPα, and p-FoxO1 compared with male counterparts (Fig. 2*H*). On LPS stimulation, male and female mutants similarly showed activation of these transcription factors. Taken together, myeloid-FATP4 deficiency led to proinflammation as observed by BMDM activation of chemokine MCP-1, proinflammatory cytokines, and inflammatory transcription factors observed in females at basal conditions, and in both males and females after LPS stimulation.

### Kupffer Cells from Fatp4^M−/−^ Mice Show an Exaggeration of Inflammatory Cytokines and Transcription Factors

By using the cell-specific Cre-LoxP recombination system, *LysM-Cre*-specific Fatp4 deletion in myeloid cells of an Fatp4^M−/−^ mouse occurs in the embryo already (38). We surmise that Fatp4 deletion may alter the composition of erythro-myeloid progenitors in the embryonic yolk sac. These progenitors can migrate, colonize the fetal liver, and give rise to fetal liver-resident MΦ or Kupffer cells (39). We investigated whether Kupffer cells could be activated by myeloid-FATP4 deficiency in a similar manner as BMDMs (Fig. 2).

**Figure 3.**
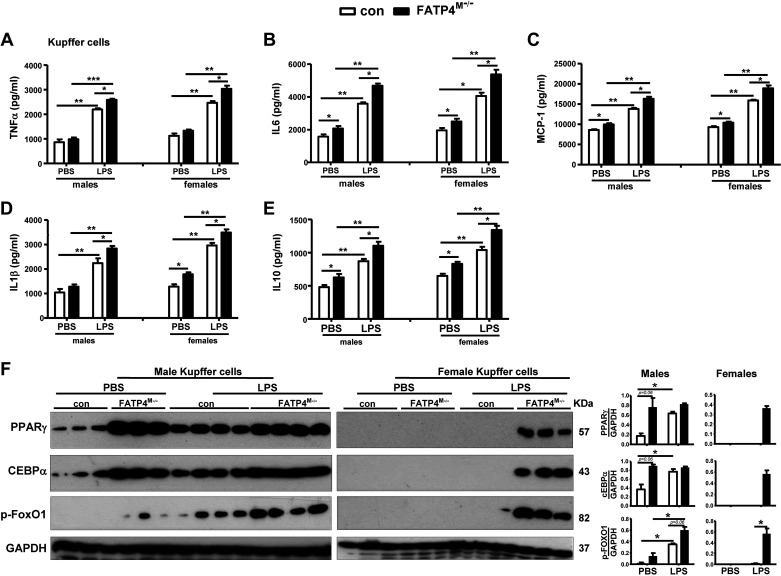
Kupffer cells from Fatp4^M−/−^ mice show elevated inflammatory cytokines and transcription factors. Kupffer cells from male and female control (Con) and Fatp4^M−/−^ mice were treated with PBS or 100 ng/mL LPS for 24 h. The release (pg/mL) of TNFα (*A*), IL6 (*B*), MCP-1 (*C*), IL1β (*D*), and IL10 (*E*) by PBS- or LPS-treated Kupffer cells from male and female mice. *F*: Western blot analysis (*left*) and quantification (*right*) of PPARγ, CEBPα, and p-FoxO1 in PBS- or LPS-treated Kupffer cells from male and female mice. Data are means ± SE, *n* = 5 (*A***–***E*) and *n* = 3 or 4 (*F*). *P < 0.05, **P < 0.01, and ***P < 0.001 by Mann-Whitney or Kruskal-Wallis tests. LPS, lipopolysaccharide.

Kupffer cells were isolated from control (Con) and Fatp4^M−/−^ mice and treated with PBS or LPS for 24 h. Kupffer cells from male and female mutants showed a similar increase in TNFα, IL6, MCP-1, IL1β, and IL10 release after LPS stimulation (Fig. 3, *A–E*). At basal conditions, Kupffer cells from male and female mutants did not show any changes in TNFα release (Fig. 3*A*). Although male mutants showed an elevated release of IL6 (Fig. 3*B*), MCP-1 (Fig. 3*C*), and IL10 (Fig. 3*E*), female mutants showed an elevated release of these cytokines and also IL1β (Fig. 3*D*) at basal conditions. In response to LPS stimulation, Kupffer cells from male and female mutants displayed elevated release of these cytokines, and with higher levels in female compared with male mutants.

On analysis of inflammatory transcription factors, Kupffer cells from male mutants displayed a trend increase in PPARγ, CEBPα, and p-FoxO1 activation at basal conditions whereas those from female mice did not show any detectable signals (Fig. 3*F*). On LPS stimulation, Kupffer cells from male control mice showed activation of these transcription factors, and a further trend activation of PPARγ and p-FoxO1 could be observed in male mutants. However, Kupffer cells from female mutants displayed a marked activation of these three transcription factors to levels comparable to male counterparts. Thus, myeloid-FATP4 deficiency in Kupffer cells supported proinflammatory response with increased cytokine release and LPS-dependent activation of PPARγ, CEBPα, and p-FoxO1 in both males and females.

### Fatp4^M−/−^ Mice Show Alterations in Body and Tissue Weights, Liver Enzymes, and Blood Lipids after HFHC Feeding

**Figure 4.**
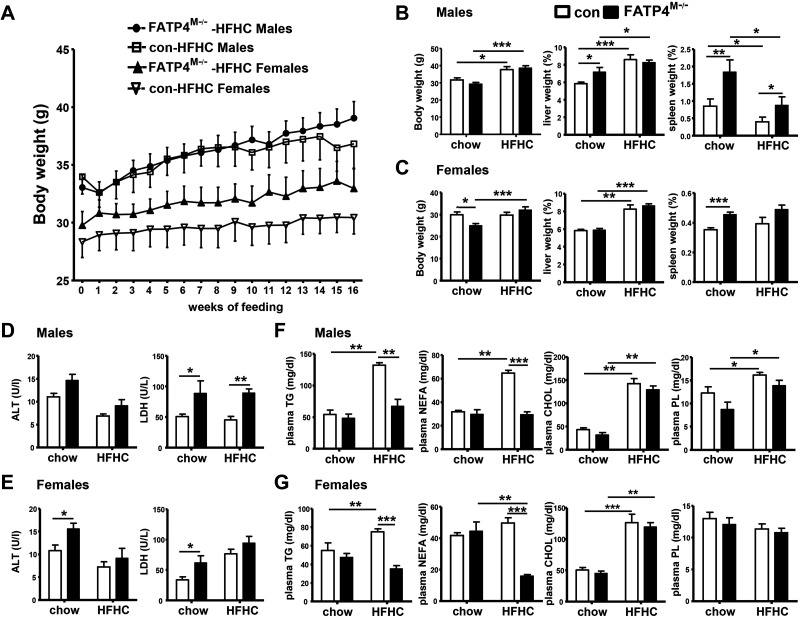
Gross phenotypic changes in body/tissue weights and blood parameters of control and Fatp4^M−/−^ mice after HFHC feeding. Male and female control (Con) and Fatp4^M−/−^ mice were fed with chow or HFHC diet for 16 wk. *A*: body weights in grams of male Con and Fatp4^M−/−^ as well as female Con and Fatp4^M−/−^ on HFHC feeding for 16 wk. Body weights (g), % liver, and % spleen (weights/body weights) of male (*B*) and female (*C*) mice fed with chow or HFHC. Plasma ALT and LDH (U/L) of male (*D*) and female (*E*) mice fed with chow or HFHC. Plasma TG, NEFA, CHOL, and PL (mg/dL) of male (*F*) and female (*G*) mice fed with chow or HFHC. Data are means ± SE, *n* = 7–10 (*A*–*C*), *n* = 5–8 (*D* and *E*), and *n* = 6–8 (*F* and *G*). *P < 0.05, **P < 0.01, and ***P < 0.001 by Mann-Whitney *U* tests. ALT, alanine transaminase; CHOL, cholesterol; HFHC, high-fat, high-cholesterol; LDH, lactate dehydrogenase; NEFA, nonesterified fatty acids; PL, phospholipids; TG, triglycerides.

We further investigated the response of Fatp4^M−/−^ mice to NASH pathogenesis on feeding with atherogenic Paigen HFHC diet. HFHC feeding led to a moderate increase in body weights in male and female control and Fatp4^M−/−^ mice (Fig. 4*A*). Myeloid-FATP4 deficiency however did not alter body weights and liver weights after 16 wk of HFHC feeding in males (Fig. 4*B*) and females (Fig. 4*C*). Notably, male and female mutants under chow already showed splenomegaly (Fig. 4, *B* and *C*), which was persistent in male mutants under HFHC feeding (Fig. 4*B*). In our model, HFHC feeding of male (Fig. 4*D*) and female (Fig. 4*E*) control mice did not markedly elevate plasma ALT and LDH activities. However, male Fatp4^M−/−^ mice under chow or HFHC already showed a significant increase in plasma LDH, whereas female mutants under chow also showed elevated ALT and LDH activities.

We further measured blood lipids of our mouse cohort. HFHC feeding of control mice caused a significant elevation of plasma TG, nonesterified fatty acids (NEFA), and cholesterol (CHOL; Fig. 4, *F* and *G*). The elevation of plasma PLs was seen only in male control mice fed with HFHC. Remarkably, under HFHC, male and female Fatp4^M−/−^ mice showed a significant attenuation in plasma TG and NEFA levels. This attenuation indicated FA mobilization and/or suppressed lipid secretion between liver and blood in mutants. Taken together, myeloid-FATP4 deficiency in male and female mice led to abnormalities in splenomegaly and liver injury independent of HFHC, and the deficiency led to suppression of blood TGs and NEFAs after HFHC feeding.

### Fatp4^M−/−^ Mice Show a Shift from TNFα to Proinflammatory and Alternatively Activated Cytokines in Blood after HFHC Feeding

**Figure 5.**
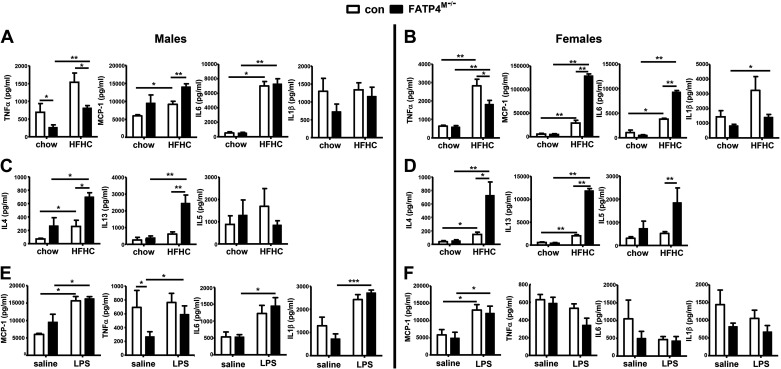
Fatp4^M−/−^ mice show elevation of MCP-1 and proinflammatory cytokines in plasma after HFHC feeding with a greater extent in females than males. Male and female control (Con) and Fatp4^M−/−^ mice were fed HFHC for 16 wk as described in Fig. 4 or intraperitoneally injected with saline or LPS (1 mg/kg and 0.5 mg/kg LPS for males and females, respectively) for 16 h. Plasma MCP-1, TNFα, IL6, and IL1β (pg/mL) of male (*A*) and female (*B*) mice fed with chow or HFHC. Plasma IL4, IL13, and IL5 (pg/mL) of male (*C*) and female (*D*) mice fed with chow or HFHC. Plasma MCP-1, TNFα, IL6, and IL1β (pg/mL) of male (*E*) and female (*F*) mice treated with saline or LPS. Data are means ± SE, *n* = 4–8 (*A*–*D*) and *n* = 4–10 (*E* and *F*). *P < 0.05, **P < 0.01, and ***P < 0.001 by Mann-Whitney *U* tests. HFHC, high-fat, high-cholesterol; MCP-1, monocyte chemoattractant protein-1.

To corroborate with blood lipids, we here analyzed MCP-1 and proinflammatory cytokines in plasma samples of our mouse cohort. Under chow, male (but not female) mutants showed a decrease in plasma TNFα (Fig. 5*A*). Under HFHC, male (Fig. 5*A*) and female (Fig. 5*B*) mutants showed an attenuation of HFHC-induced TNFα. On the contrary, HFHC-induced MCP-1 was further elevated in male and female mutants by ∼0.6- and ∼3-folds, respectively. HFHC-fed female mutants also showed further elevation of IL6 by ∼1.6-folds. For alternatively activated cytokines, HFHC feeding of control mice caused an elevation of IL4 in males (Fig. 5*C*) and females (Fig. 5*D*), and additionally IL13 in females. HFHC-fed male mutants showed a further increase in IL4 and IL13 by approximately two- to threefolds (Fig. 5*C*), whereas female counterparts showed a further increase in these cytokines as well as IL5 by approximately three- to fourfolds (Fig. 5*D*). Thus, myeloid-FATP4 deficiency under HFHC led to a shift from TNF-α toward MCP-1 in males and females, and a shift toward MCP-1, IL6, IL4, IL13, and IL5 was greater in females than males.

It has been shown that an increase in Cer by myeloid-specific deletion of sphingosine kinases did not render mice susceptible to LPS-induced injury in vivo (40). Since our mutant BMDMs showed suppressed Cer levels (Fig. 2, *C* and *D*), we investigated how our mutants would respond to LPS in vivo. In this cohort, male mice were intraperitoneally treated with 1 mg/kg LPS, whereas female mice were treated with 0.5 mg/kg LPS overnight. LPS treatment of control mice caused an elevation of plasma MCP-1 in both males (Fig. 5*E*) and females (Fig. 5*F*). However, the levels of plasma MCP-1, TNFα, IL6, and IL1β (Fig. 5, *E* and *F*), as well as white blood cells (data not shown), were not altered in both male and female mutants. Our results were thus consistent with the previous report (40). Taken together, myeloid-FATP4 deficiency exacerbated proinflammatory and alternatively activated cytokines during HFHC-induced NASH, but not during acute LPS in vivo.

### Fatp4^M−/−^ Mice under HFHC Feeding Show Exaggerated Hepatic Steatosis in Males and Immune Cell Infiltration in Females

**Figure 6.**
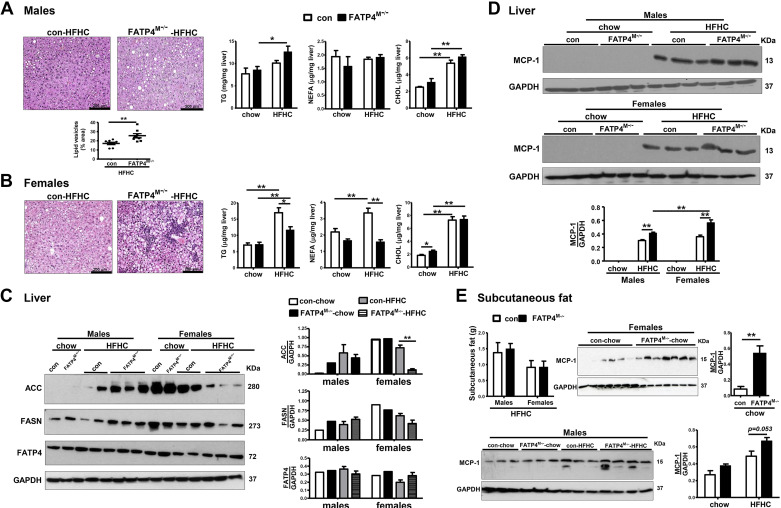
Fatp4^M−/−^ mice show increased exacerbation of HFHC-induced NASH with a greater inflammatory response in females than males. Male and female control (Con) and Fatp4^M−/−^ mice were under chow or HFHC as described in Fig. 4. *A*: *left*, representatives of H&E-stained liver sections (*top*) and liver lipid vesicles in % area (*bottom*) from male mice fed with HFHC. *Right*: levels of liver TG, NEFA, and CHOL (μg/mg liver) of male mice fed with chow or HFHC. *B*: *left*: representatives of H&E-stained liver sections from female mice fed with HFHC. *Right*: levels of liver TG, NEFA, and CHOL (μg/mg liver) of female mice fed with chow or HFHC. Western blot analysis (*left*) and quantification (*right*) of ACC, FASN, and FATP4 (*C*) and MCP-1 (*D*) in livers of male and female mice fed with chow or HFHC. *E*: subcutaneous fat weights (g) of male and female mice fed with HFHC (*top*, *left*) and MCP-1 expression in subcutaneous fat of female mice fed with chow (*top*, *right*). *Bottom*: MCP-1 expression in subcutaneous fat of male mice fed with chow or HFHC. Data are means ± SE, *n* = 6–8 (*A*, *B*, and *D*), *n* = 3–7 (*C* and *E*). *P < 0.05 and **P < 0.01 by Mann-Whitney *U* tests. CHOL, cholesterol; Fatp4^M−/−^, macrophage-specific Fatp4-deficient mice; HFHC, high-fat, high-cholesterol; MCP-1, monocyte chemoattractant protein-1; NASH, nonalcoholic steatohepatitis; NEFA, nonesterified fatty acids; TG, triglycerides.

We further characterized the livers and subcutaneous fat of our mouse cohort. On histological evaluation, HFHC feeding of control mice induced only a few lipid vesicles seen in livers of males (Fig. 6*A*) and females (Fig. 6*B*). Image analyses revealed that HFHC-fed male mutants showed a significant increase in hepatic lipid vesicles (Fig. 6*A*). However, every HFHC-fed female mutants showed massive presence of hepatic inflammatory cells (Fig. 6*B*), which in turn hindered an accurate evaluation of hepatic lipid vesicles by image analysis. The analyses of liver lipids showed that HFHC feeding of control mice significantly increased the levels of hepatic CHOL in males (Fig. 6*A*) and TG, NEFA, and CHOL in females (Fig. 6*B*). Consistent with histology (Fig. 6*A*), a further trend elevation of hepatic TG could be observed in HFHC-fed male mutants. On the contrary, HFHC-fed female mutants showed a significant decrease in liver TG and NEFA levels (Fig. 6*B*).

To determine whether there was a defect in de novo FA synthesis in HFHC-fed female mutants, we analyzed protein expression of acetyl-CoA carboxylase (ACC, converting acetal-CoA to malonyl-CoA) and fatty acid synthase (FASN, converting malonyl-CoA to palmitate). Compared with HFHC-fed controls, the expression of ACC was significantly attenuated in HFHC-fed female mutants, and only a trend attenuation could be observed for FASN expression (Fig. 6*C*). This was not the case for male mutants fed with HFHC. Moreover, hepatic FATP4 expression was not markedly altered in any male and female mutants fed with HFHC. Thus, the observed changes in hepatic lipids were not correlated with hepatic FATP4 expression. The reduction of hepatic TG and NEFA in female mutants fed with HFHC was due to suppression of de novo FA synthesis.

To correlate with MCP-1 activation in mutant BMDMs (Fig. 2*G*), we determined MCP-1 expression not only in the liver but also in subcutaneous fat in our mouse cohort, because inflammation in adipose tissues during NASH is shown to be mediated by MCP-1/CCR2 pathway (41). HFHC feeding of control mice induced an increase in hepatic MCP-1 protein expression (Fig. 6*D*). Under HFHC, mutants showed an exaggerated expression in males and females by ∼33% and ∼52%, respectively. No changes in subcutaneous fat weights could be observed among control and mutant mice fed with HFHC (Fig. 6*E*). However, MCP-1 protein expression was markedly upregulated in chow-fed female mutants and with a trend in HFHC-fed male mutants. Thus, myeloid-FATP4 deficiency under HFHC led to exacerbation of NASH with increased hepatic steatosis in males and hepatic inflammatory response in females. The latter could be supported by greater liver and subcutaneous fat expression of MCP-1 in females compared with male counterparts.

### Fatp4^M−/−^ Mice Show Exacerbation of Hepatic Fibrosis after HFHC Feeding with a Greater Response in Females than Males

**Figure 7.**
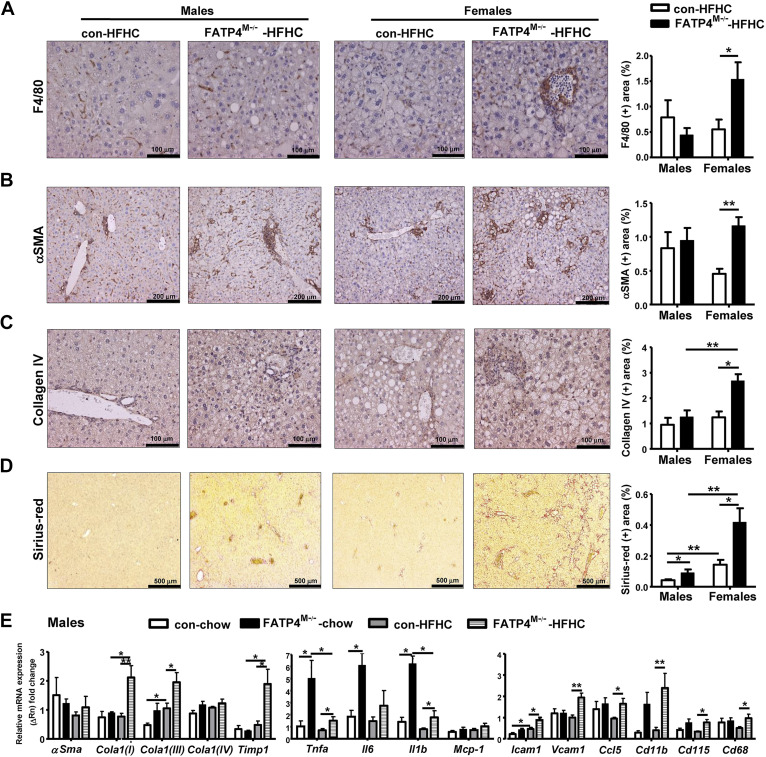
Fatp4^M−/−^ mice show increased hepatic inflammatory fibrosis after HFHC feeding with a greater response in females than males. Male and female control (Con) and Fatp4^M−/−^ mice were under chow or HFHC as described in Fig. 4. Representatives of F4/80-IHC (*A*), αSMA-IHC (*B*), collagen IV IHC (*C*), and Sirius-red (+) (*D*) in stained liver sections (*left*) and quantification as % (+) area (*right*) of male and female mice fed with HFHC. *E*: hepatic mRNA expression of fibrosis genes *αSma, Cola1(I), Cola1(III), Cola1(IV)*, and *Timp1* as well as proinflammatory genes *Tnfa, Il6, Il1b, Mcp-1*, *Icam1, Vcam1, Ccl5, Cd11b*, *Cd115*, and *Cd68* in male mice fed with chow or HFHC. Data are means ± SE, *n* = 6–8 (*A*, *B*, and *D*), and *n* = 4–7 (*C* and *E*) *P < 0.05 and **P < 0.01 by Mann-Whitney *U* tests. Fatp4^M−/−^, macrophage-specific Fatp4-deficient mice; HFHC, high-fat, high-cholesterol; IHC, immunohistochemistry.

To associate with hepatic MCP-1 and infiltration of immune cells, IHC analysis of granulocyte marker F4/80 was performed in livers of our mouse cohort. Results showed that the percentages of F4/80 (+) cells were increased in female but not male Fatp4^M−/−^ mice fed with HFHC (Fig. 7*Α*). For the analysis of liver fibrosis, we performed IHC of myofibroblast marker α-smooth muscle actin (α-SMA), collagen IV, and Sirius-red staining. No changes in the percentages of α-SMA (+) cells could be observed in HFHC-fed male mutants, whereas HFHC-fed female mutants showed a marked increase by ∼2.6 folds (Fig. 7*B*). Similarly, an increase in the percentages of collagen IV (+) cells (Fig. 7*C*) and Sirius-red (+) staining (Fig. 7*D*) was observed in female mutants. Collagen IV was present in the basement membranes under endothelial cells of the portal vein and hepatic arteries of male and female control mice fed with HFHC (Fig. 7*C*). However, female mutants fed with HFHC showed an intense staining of collagen IV around small vessels of the portal tracts that displayed inflammatory cell infiltration and along the wall of adjacent sinusoids.

To further evaluate hepatic fibrosis in male mice, we analyzed mRNA expression of inflammation and fibrosis genes in their livers. HFHC feeding of control mice induced a moderate upregulation of *Cola1 (III)* and *Icam1* (Fig. 7*E*). Remarkably, HFHC-fed mutants showed a significant upregulation of various inflammatory markers including fibrosis *Col1a1 (I)*, *Cola1 (III)*, and *Timp-1*; inflammatory cytokines *Tnfa* and *Il1b*; and chemokines and immune cells *Icam1*, *Vcam1*, *Ccl5*, *Cd11b*, *Cd115*, and *Cd68*. Notably, male mutants under chow already showed a significant upregulation of *Tnfa*, *Il6*, and *Il1b* mRNA expression, which was consistent with elevation of LDH (Fig. 4*D*). Thus, male Fatp4^M−/−^ mice showed basal hepatic inflammation under chow and aggravated hepatic fibrosis on HFHC feeding but with a lesser extent than female counterparts.

## DISCUSSION

FATP4 plays a major role in skin physiology in mice (5–7) and humans (8), and patients with FATP4 mutations manifest allergies and eosinophilia (9) implying a pivotal role of FATP4 on MΦ functions. FATP4 is also associated with adiposity, insulin resistance, and blood lipids (16–18), and this notion can be supported by our previous studies in adipose- or hepatocyte-specific Fatp4-deficient mice (14, 15). Our current study demonstrated the role of myeloid FATP4 on NASH pathogenesis. During HFHC-induced NASH, plasma MCP-1, and proinflammatory cytokines as well as expression of hepatic MCP-1 and fibrosis markers were markedly elevated to a greater extent in female than male mutants. Thus, our results provide new insights into a sexually dimorphic role of myeloid-FATP4 on inflammatory NASH.

Under chow diet, Fatp4^M−/−^ mice displayed hepatomegaly (Fig. 4*B*) and hepatic inflammation (Fig. 7*E*) in males, and subcutaneous fat inflammation in females (Fig. 6*E*). Both male and female mutants under chow showed thrombocytopenia (Fig. 1*B*), splenomegaly (Fig. 4, *B* and *C*), and elevation of liver enzymes (Fig. 4, *D* and *E*). Thus, myeloid-FATP4 deficiency elicited abnormalities not only in the liver but also systemically. The observed thrombocytopenia could be due to an immaturity of mini-megakaryocytes in the bone marrow (29) and/or altered lipids in the plasma membranes of platelets (42). As altered SM can affect hemostatic and thrombotic processes (43), these events may occur as a result of a decrease in platelet counts by myeloid-FATP4 deficiency. As corneocytes located in the stratum corneum contain 50% Cer, FATP4 and β-glucocerebrosidase (*GBA1*) are among the proteins identified to be involved in the formation of Cer-bound envelope of corneocytes (44). Notably, the decrease of Cer caused by *GBA1* mutation leads to the development of Type-2 Gaucher disease (or collodion babies). These patients exhibit hepatosplenomegaly, and their Gaucher cells reportedly resemble alternatively activated MΦ (45). Similar to Gaucher cells, the hyperplasia of Fatp4^M−/−^ myeloid cells could result in hepatomegaly in males and splenomegaly in males and females. The significance of Cer metabolism in MΦ is clearly seen in IPS (5–8) and Gaucher disease (44, 45), the alteration of SPLs and PLs in FATP4-deficient myeloid cells may thus likely affect MΦ inflammatory response.

We demonstrated that mutant BMDMs showed the reduction of Cer and SM in both males and females, and BMDMs from female mutants additionally showed a significant reduction of PC, PE, and PI. In addition to VLFA-Cer being protective in MΦ (30, 31), the suppressed Cer species may allow activation of the M2-regulator PPARγ (46) resulting in elevation of basal IL4 and IL13 release by mutant BMDMs. Strikingly, BMDM release of LPS-stimulated MCP-1 and proinflammatory cytokines as well as expression of MCP-1, PPARγ, CEBPα, and p-FoxO1 were further increased in mutants, and with a greater extent in females than males. As SM and PLs are required for the maintenance of plasma membrane fluidity (47) and MΦ functions (48), their reduction in female mutants may consistently lead to a greater proinflammatory response (49). It is shown that saturated-Cer are used for SM synthesis whereas unsaturated Cer are hydrolyzed to generate PUFAs, which are then channeled for synthesis of PLs (50). The latter PL synthesis would likely be preferential in females because estrogen is shown to target PL synthesis (51), and it also regulates inflammatory severity via PPARγ (52). As Kupffer cells come from yolk sac (39) and bone-marrow-derived monocytes shortly after birth (53), it is conceivable that these Kupffer cells from mutant mice can display overall proinflammatory events in a similar manner as their BMDMs. It has been suggested that Kupffer cells dominate the hepatic MΦ pool in homeostasis and bone-marrow-derived monocytes respond to liver injury (54). Such dichotomous (protective and detrimental) immune activation could be observed in HFHC-fed Fatp4^M−/−^ mice, as they showed an attenuation of TNFα but elevation of MCP-1, IL6, IL4, IL13, and IL5 in plasma. On HFHC feeding, injured hepatocytes may induce the release MCP-1 which triggers an egress of monocytes from bone marrow. FATP4-deficient monocyte-derived Kupffer cells (53) and bone-marrow-derived monocytes (54) with elevated MCP-1 and inflammatory cytokines may further perpetuate hepatic inflammation and fibrosis.

We observed that female Fatp4^M−/−^ mice respond to HFHC feeding showing the elevation of MCP-1, IL6, IL4, IL13, and IL5, thus indicating a greater response toward alternatively activated MΦ compared with male counterparts. Concurrently, type 2 immunity (IL10 and IL4) has been linked to progression to NASH, and the elevation of plasma IL4, IL5, and IL13 is reported in patients with NASH (55). Moreover, IL13 is shown to activate ductular reaction, steatosis, and fibrosis (56). The striking greater response in female mutants could be the effects of estrogen on M2/Th2 activation reported in asthma (57) and NAFLD (58). The shift toward alternatively activated MΦ in HFHC-fed mutants may likely be related to suppressed levels of MΦ Cer, SM, and PLs, and the suppression of PLs was more predominant in females. These conditions at the MΦ levels however did not render mutant mice susceptible to acute LPS in vivo (Fig. 5, *E* and *F*). This could be due to the metabolic differences between exogenous (NASH) and endogenous (LPS in vivo) sources of FAs for lipid synthesis in MΦ. Although NASH is associated with suppressed hepatic PL synthesis (59), however, LPS activates PL synthesis necessary for cytokine production (60). Thus, the further suppression of MΦ PLs by myeloid-FATP4 deficiency may likely exacerbate the pathogenesis of NASH, but not LPS in vivo.

It is known that males are more prone to HFHC-induced hepatic steatosis (61), whereas estrogen protects against hepatic steatosis (62). Here, myeloid-FATP4 deficiency may promote male-prone abnormalities by exacerbating hepatic steatosis possibly via the strong activation of PPARγ, CEBPα, and p-FoxO1 in their Kupffer cells (Fig. 3*F*). It is shown that systemic inflammation can suppress the release of lipoproteins (63), and the observed attenuated levels of plasma TG and NEFA could also result in increased hepatic steatosis observed in male mutants. We also demonstrated that hepatic expression of de novo FA synthesis gene ACC was severely downregulated in female mutants fed with HFHC. Consistently, ACC is a known target of estrogen (64). Moreover, severe inflammation in these mutants can in turn inhibit hepatic de novo FA biosynthesis (65) thus resulting in a decrease in TG and NEFA levels in their livers and plasma. Thus, our results demonstrated sex-bias susceptibility to NASH by myeloid-FATP4 deficiency via alteration of lipid metabolism in liver and systemically.

Regarding other FATP members, forced expression of FATP1 in THP-1 cells increases Cer levels (20). Thus, FATP1 provides acyl-CoAs for Cer synthesis as well. Global deletion of FATP1 in mice leads to increased adiposity and worsened metabolic syndrome on high-fat-diet feeding (21). Myeloid-specific Fatp1 deletion in Ldlr^−/−^ mice results in exaggeration of atherosclerosis (66). In contrast to FATP1 and FATP4, FATP2 knockdown in vivo improves hepatosteatosis (67). Unlike FA-uptake mechanism of FATP2, the syntheses of SPLs and PLs regulated by FATP4 or FATP1 play a pivotal role in myeloid functions as their deficiency worsens NASH. It is shown that suppressed Cer can lead to cognitive dysfunctions in Gaucher disease (68). Myeloid suppression of Cer due to FATP4 mutations may lead to certain brain abnormalities, such as hereditary Melkersson-Rosenthal syndrome with facial paralysis, which is linked to FATP1 mutations (69).

The significance of our results may be relevant to the manifestation of M2/Th2-related allergies and eosinophilia seen in adult IPS patients with FATP4 mutations (9). Female patients may particularly be at risk of hepatic fibrosis when consuming HFHC-rich diets. IPS is a rare disease commonly seen in Sweden and Norway, and some cases are reported in other European countries, Africa, and Asia. On our review of case reports, eosinophilia was reported to be transient in four baby boys (70–73) and a baby girl (74) with IPS and FATP4 mutations. However, persistent eosinophilia was reported in two female patients with IPS at the age of 14 (75) and 27 (76). This age- and sex-specific eosinophilia resembles the abnormalities seen in M2-related diseases including asthma (57) and allergies (77), which are predominantly seen in boys before puberty and this sex preference reverses after puberty. Moreover, it is proposed that FATP4 mutations could result in barrier defective permeability in atopic dermatitis associated with suppressed skin VLCFA-Cer levels (78). A higher prevalence of atopic dermatitis is also found in women due to the greater promotion of type-2 immunity by female hormones (79). Hence, eosinophilia and HFHC-induced hepatic fibrosis may be prevalent in adult females with FATP4 mutations.

In conclusion, myeloid-FATP4 deficiency led to the reduction of BMDM SPLs and PLs in BMDMs. Proinflammatory LPS response was observed in BMDMs and Kupffer cells of Fatp4^M−/−^ mice. Such activation was associated with thrombocytopenia, splenomegaly, and liver injury in mutant mice under chow diet. During HFHC-induced NASH, male mutants exhibited exaggerated hepatic steatosis whereas female mutants exhibited aggravated hepatic inflammatory fibrosis. Our study demonstrated a sex-dimorphic role of myeloid-FATP4 on steatotic versus inflammatory NASH, and provided insights into strategic considerations for NASH treatment in sex-specific personalized therapies.

## DATA AVAILABILITY

Data will be made available on reasonable request.

## GRANTS

W.C. and T.H. acknowledge the funding from Deutsche Forschungsgemeinshaft (CH 288/6-2, HE 5521/1-1). M.U.M. acknowledges the funding from Deutsche Forschungsgemeinshaft SFB1118-FerrOs-FOR5146 and schwerpunktprogramm MU1108/9-1.

## DISCLAIMERS

The funding sources had no involvement in study design; the collection, analysis, and interpretation of data; writing of the report; and the decision to submit the article for publication.

## DISCLOSURES

No conflicts of interest, financial or otherwise, are declared by the authors.

## AUTHOR CONTRIBUTIONS

U.M. and W.C. conceived and designed research; D.G., C.J., Y.Z., S.S., S.T-K., and S.A. performed experiments; D.G., C.J., Y.Z., S.S. S.A., and W.C. analyzed data; D.G., T.H., and W.C. interpreted results of experiments; D.G. and C.J. prepared figures; D.G. drafted manuscript; D.G., C.J., M.U.M., U.M., T.H., and W.C. edited and revised manuscript; D.G., C.J., M.U.M., U.M., T.H., and W.C. approved final version of manuscript.
